# Comparative Metabolomics and Transcriptomics Reveal Multiple Pathways Associated with Polymyxin Killing in Pseudomonas aeruginosa

**DOI:** 10.1128/mSystems.00149-18

**Published:** 2019-01-08

**Authors:** Mei-Ling Han, Yan Zhu, Darren J. Creek, Yu-Wei Lin, Alina D. Gutu, Paul Hertzog, Tony Purcell, Hsin-Hui Shen, Samuel M. Moskowitz, Tony Velkov, Jian Li

**Affiliations:** a Biomedicine Discovery Institute, Infection and Immunity Program, Department of Microbiology, Monash University, Clayton, Victoria, Australia; b Drug Delivery, Disposition and Dynamics, Monash Institute of Pharmaceutical Sciences, Monash University, Parkville, Victoria, Australia; c Department of Molecular Biology, Massachusetts General Hospital, Boston, Massachusetts, USA; d Centre for Innate Immunity and Infectious Diseases, Monash Institute of Medical Research, Monash University, Clayton, Victoria, Australia; e Department of Biochemistry and Molecular Biology, Monash University, Clayton, Victoria, Australia; f Department of Materials Science and Engineering, Faculty of Engineering, Monash University, Clayton, Victoria, Australia; g Vertex Pharmaceuticals, Boston, Massachusetts, USA; h Department of Pharmacology & Therapeutics, School of Biomedical Sciences, Faculty of Medicine, Dentistry and Health Sciences, The University of Melbourne, Parkville, Victoria, Australia; University of California, Berkeley

**Keywords:** lipid A modification, metabolomics, *Pseudomonas aeruginosa*, transcriptomics, glycerophospholipids, lipopolysaccharide, polymyxins

## Abstract

Polymyxins are a last-line therapy against multidrug-resistant Pseudomonas aeruginosa; however, resistance to polymyxins has been increasingly reported. Therefore, understanding the mechanisms of polymyxin activity and resistance is crucial for preserving their clinical usefulness. This study employed comparative metabolomics and transcriptomics to investigate the responses of polymyxin-susceptible P. aeruginosa PAK (polymyxin B MIC, 1 mg/liter) and its polymyxin-resistant *pmrB* mutant PAK*pmrB6* (MIC, 16 mg/liter) to polymyxin B (4, 8, and 128 mg/liter) at 1, 4, and 24 h, respectively. Our results revealed that polymyxin B at 4 mg/liter induced different metabolic and transcriptomic responses between polymyxin-susceptible and -resistant P. aeruginosa. In strain PAK, polymyxin B significantly activated PmrAB and the mediated *arn* operon, leading to increased 4-amino-4-deoxy-L-arabinose (L-Ara4N) synthesis and the addition to lipid A. In contrast, polymyxin B did not increase lipid A modification in strain PAK*pmrB6*. Moreover, the syntheses of lipopolysaccharide and peptidoglycan were significantly decreased in strain PAK but increased in strain PAK*pmrB6* due to polymyxin B treatment. In addition, 4 mg/liter polymyxin B significantly perturbed phospholipid and fatty acid levels and induced oxidative stress in strain PAK, but not in PAK*pmrB6*. Notably, the increased trehalose-6-phosphate levels indicate that polymyxin B potentially caused osmotic imbalance in both strains. Furthermore, 8 and 128 mg/liter polymyxin B significantly elevated lipoamino acid levels and decreased phospholipid levels but without dramatic changes in lipid A modification in wild-type and mutant strains, respectively. Overall, this systems study is the first to elucidate the complex and dynamic interactions of multiple cellular pathways associated with the polymyxin mode of action against P. aeruginosa.

**IMPORTANCE** Pseudomonas aeruginosa has been highlighted by the recent WHO Global Priority Pathogen List due to multidrug resistance. Without new antibiotics, polymyxins remain a last-line therapeutic option for this difficult-to-treat pathogen. The emergence of polymyxin resistance highlights the growing threat to our already very limited antibiotic armamentarium and the urgency to understand the exact mechanisms of polymyxin activity and resistance. Integration of the correlative metabolomics and transcriptomics results in the present study discovered that polymyxin treatment caused significant perturbations in the biosynthesis of lipids, lipopolysaccharide, and peptidoglycan, central carbon metabolism, and oxidative stress. Importantly, lipid A modifications were surprisingly rapid in response to polymyxin treatment at clinically relevant concentrations. This is the first study to reveal the dynamics of polymyxin-induced cellular responses at the systems level, which highlights that combination therapy should be considered to minimize resistance to the last-line polymyxins. The results also provide much-needed mechanistic information which potentially benefits the discovery of new-generation polymyxins.

## Abstract (precis)

Pseudomonas aeruginosa has been highlighted by the recent WHO Global Priority Pathogen List due to multidrug resistance. Without new antibiotics, polymyxins remain a last-line therapeutic option for this difficult-to-treat pathogen. The emergence of polymyxin resistance highlights the growing threat to our already very limited antibiotic armamentarium and the urgency to understand the exact mechanisms of polymyxin activity and resistance. Integration of the correlative metabolomics and transcriptomics results in the present study discovered that polymyxin treatment caused significant perturbations in the biosynthesis of lipids, lipopolysaccharide, and peptidoglycan, central carbon metabolism, and oxidative stress. Importantly, lipid A modifications were surprisingly rapid in response to polymyxin treatment at clinically relevant concentrations. This is the first study to reveal the dynamics of polymyxin-induced cellular responses at the systems level, which highlights that combination therapy should be considered to minimize resistance to the last-line polymyxins. The results also provide much-needed mechanistic information which potentially benefits the discovery of new-generation polymyxins.

## INTRODUCTION

Pseudomonas aeruginosa is a prominent opportunistic pathogen that can cause chronic lung infections in cystic fibrosis patients, as well as serious acute infections in immunocompromised and injured individuals (1). However, due to its highly intrinsic and adaptive resistance to a wide range of antibiotics, infections caused by this organism are often very difficult to treat (2). For this reason, the use of polymyxins (i.e., polymyxin B and colistin) has resurged as a last-line therapeutic option over the last decade (3). Polymyxins are a family of cyclic lipopeptides that display a narrow spectrum of activity against Gram-negative bacteria (4). The putative mechanism of antibacterial killing of polymyxins involves an initial electrostatic interaction between the positively charged L-α,γ-diaminobutyric acid (Dab) residues of polymyxins and the negatively charged phosphate groups of lipid A in Gram-negative outer membrane (OM) (4, 5). This interaction results in the displacement of divalent cations (Mg^2+^ and Ca^2+^) that bridge adjacent lipid A molecules and allows the hydrophobic moieties (N-terminal fatty acyl tail and D-Phe^6^-L-Leu^7^) of polymyxins to penetrate into the OM (4, 6). The insertion acts to facilitate polymyxins to cross the OM and is believed to promote the exchange of phospholipids between the OM and inner membrane (IM), which therefore disrupts the integrity of IM phospholipids, causes osmotic imbalance, and consequently leads to cell death (7, 8). Another proposed mechanism suggests that polymyxins induce bacterial cell death through the formation of hydroxyl radicals, leading to the oxidative damage of DNA, lipids, and proteins (9, 10). However, the precise cellular mechanism of polymyxin activity has not been well defined.

The most common mechanism of polymyxin resistance is through covalent modifications of lipid A phosphate groups with positively charged moieties, such as 4-amino-4-deoxy-L-arabinose (L-Ara4N) and/or phosphoethanolamine (pEtN), which decrease the net negative charge of lipid A and repel the binding to positively charged polymyxins (11). Moreover, bacteria are able to attain high-level polymyxin resistance by both charge-charge repulsion and hydrophobic alterations of lipid A (12). In P. aeruginosa, polymyxin resistance is mainly due to lipid A modification with L-Ara4N which is mediated by mutations in several two-component regulatory systems (TCRs) (e.g., PhoPQ, PmrAB, and/or ParRS) and the subsequent constitutive expression of the *arnBCADTEF-pmrE* operon (13–15). Although the incidence of polymyxin resistance in the clinic is relatively low, suboptimal dosing and increased use of polymyxins may significantly increase the resistance (16). More worryingly, the recent emergence of plasmid-mediated polymyxin resistance via *mcr* genes implies that polymyxin resistance may readily spread (17). Therefore, there is a clear unmet need for the discovery of novel alternative drug targets against polymyxin resistance.

To date, systems pharmacology has been increasingly employed to investigate the detailed mechanisms of antibiotic activity and resistance (18–21). In particular, metabolomics provides a powerful systems tool to define the diversity and abundance of small metabolite molecules within bacterial cells and to determine the direct consequence of bacterial responses to antibiotics (18, 19, 22). Transcriptomics enables us to interpret the functional elements of the genome and reveal the global gene expression profiles in bacterial cells (20, 23, 24). The integration of metabolomics and transcriptomics helps to investigate the functional correlations between metabolism and gene expression and to identify metabolic pathways that are essential in cellular responses to antibiotic treatment. In the present study, correlative metabolomics and transcriptomics were conducted to investigate cellular metabolic perturbations and differentially expressed genes in paired polymyxin-susceptible and -resistant P. aeruginosa strains in response to polymyxin treatment. This integrated omics approach provides detailed mechanistic insights into the mechanisms of antibacterial killing and resistance to polymyxins, as well as potential intracellular targets to tackle resistance to this last-line class of antibiotics.

## RESULTS

### Metabolic and lipidomic perturbations in response to polymyxin B.

According to the Clinical and Laboratory Standards Institute (CLSI) and European Committee on Antimicrobial Susceptibility Testing (EUCAST) guidelines (2017), the clinical resistance breakpoint of polymyxin B against P. aeruginosa is ≥4 mg/liter (25). Therefore, 4 mg/liter polymyxin B was used to examine the metabolic responses in the paired polymyxin-susceptible P. aeruginosa PAK (polymyxin B MIC, 1 mg/liter) and polymyxin-resistant PAK*pmrB6* (polymyxin B MIC, 16 mg/liter) strains at 1, 4, and 24 h (see Fig. S1 in the supplemental material). To ensure that both the hydrophilic and lipophilic metabolites were detected, hydrophilic interaction liquid chromatography (HILIC) and reversed-phase liquid chromatography (RPLC) were employed, yielding 871 (see Data Set S1 in the supplemental material) and 427 (Data Set S2) putatively identified metabolites, respectively. These metabolites were involved in a wide range of pathways, including amino acids, carbohydrates, lipids, nucleotides, and secondary metabolites. The technical performance was monitored based on periodic analysis of pooled biological quality control (PBQC) samples with the median relative standard deviation (RSD) values of 10.5% (HILIC) and 12.9% (RPLC), which were well within the acceptable limits for metabolomics (Table S1) (26). The reproducibility of metabolomics data are potentially affected by analytical techniques, sampling procedures, and natural biological variability; the median RSD values for all sample groups were between 25 and 40% (Table S1), which were generally within acceptable limits (26). Moreover, the principal-component analysis (PCA) score plots showed that the PBQC samples were clustered tightly by both methods (Fig. S2), indicating minimal analytical variation and consistency with the RSD values.

10.1128/mSystems.00149-18.1 FIG S1 Time kill kinetics of polymyxin B (PMB) against strains PAK (A) and PAK*pmrB6* (B) at 1, 4, and 24 h (*n* = 2). The concentrations of PMB were 4 and 8 mg/liter for strain PAK, while the concentrations were 4 and 128 mg/liter for strain PAK*pmrB6*. Download FIG S1, TIF file, 0.3 MB. Copyright © 2019 Han et al. 2019 Han et al. This content is distributed under the terms of the Creative Commons Attribution 4.0 International license.

10.1128/mSystems.00149-18.2 FIG S2 PCA score plots of the polymyxin B-treated and -untreated samples as well as QC samples analyzed by HILIC (A) and RPLC (B) methods. Download FIG S2, TIF file, 0.4 MB. Copyright © 2019 Han et al. 2019 Han et al. This content is distributed under the terms of the Creative Commons Attribution 4.0 International license.

10.1128/mSystems.00149-18.3 DATA SET S1 Metabolomics data set collected by the HILIC method. Download Data Set S1, XLSX file, 3.3 MB. Copyright © 2019 Han et al. 2019 Han et al. This content is distributed under the terms of the Creative Commons Attribution 4.0 International license.

10.1128/mSystems.00149-18.4 DATA SET S2 Metabolomics data set collected by the RPLC method. Download Data Set S2, XLSX file, 2.9 MB. Copyright © 2019 Han et al. 2019 Han et al. This content is distributed under the terms of the Creative Commons Attribution 4.0 International license.

10.1128/mSystems.00149-18.6 TABLE S1 Median relative standard deviation (RSD) values for all metabolites. Each data set was based on five biological replicates measured by the HILIC or RPLC method. Download Table S1, DOCX file, 0.01 MB. Copyright © 2019 Han et al. 2019 Han et al. This content is distributed under the terms of the Creative Commons Attribution 4.0 International license.

**FIG 1:**
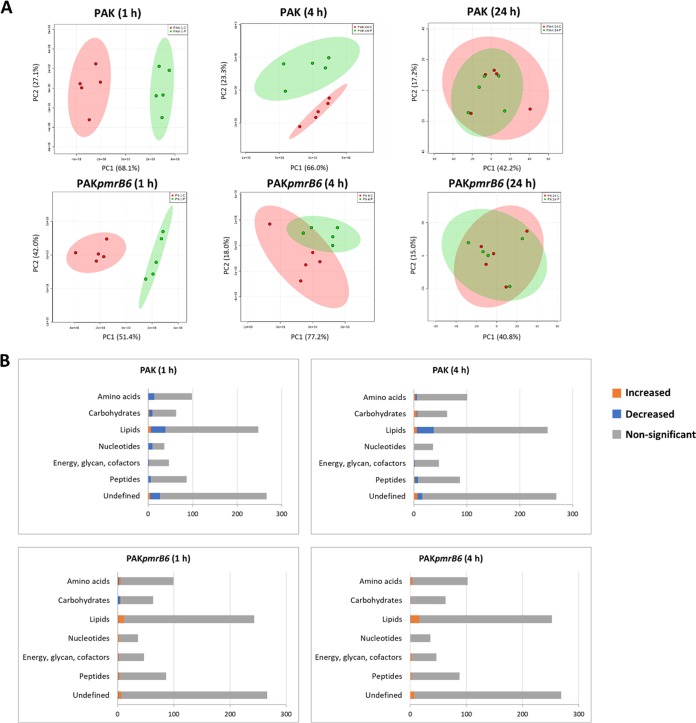
Multivariate and univariate analyses of global metabolic changes. (A) PCA score plots of the first two principal components (PC1 and PC2) for metabolite levels from *P. aeruginosa* PAK and PAK*pmrB6* treated with 4 mg/liter polymyxin B at 1, 4, and 24 h. Each data set represents a total of 10 samples containing five biological replicates of each condition. The untreated control is shown in red, and polymyxin B-treated samples are shown in green. (B) Bar charts show the number of significantly increased and decreased metabolites in the major metabolic pathways in strains PAK and PAK*pmrB6* due to 4 mg/liter polymyxin B treatment at 1 and 4 h (fold change > 2, *P* < 0.05, Student’s *t* test). The numbers of metabolites are shown on the *x* axes.

**FIG 2:**
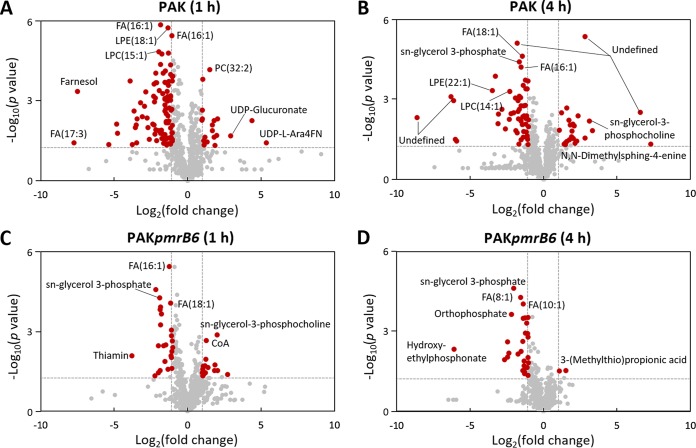
Volcano plots show the fold change and significance of metabolites in strains PAK (A and B) and PAK*pmrB6* (C and D) in response to 4 mg/liter polymyxin B at 1 and 4 h. The log_2_ fold change is shown from −10 to 10 on the *x* axis. Statistical significance displayed by −log_10_ (*P* value) is shown from 0 to 6 on the *y* axis. Metabolites having a fold change of >2 and *P* < 0.05 (Student’s *t* test) are shown by the red dots. Metabolites that were not significantly changed are shown by gray dots. Fold changes of the treated samples relative to the untreated control are based on mean values of five biological replicates in both strains.

From PCA score plots, the most significant overall metabolic change induced by polymyxin B (4 mg/liter) treatment was observed at 1 h, whereas little difference was observed at 24 h between the polymyxin-susceptible and -resistant P. aeruginosa strains. The perturbations of metabolites in the wild-type PAK strain with polymyxin B treatment at 4 h were still distinguishable, but not significant in strain PAK*pmrB6* (Fig. 1A). With regards to the number of significantly changed metabolites (fold change [FC] > 2; *P < *0.05) at 1 and 4 h, polymyxin B treatment resulted in 12.9% and 9.0% metabolic changes in the polymyxin-susceptible wild-type PAK strain, respectively, but only 4.9% and 3.7% metabolic changes in the polymyxin-resistant PAK*pmrB6* strain, respectively (Fig. 1B and 2; Data Set S3). Intriguingly, polymyxin B treatment (4 mg/liter) dramatically perturbed a wide range of key pathways, including lipids, carbohydrates, nucleotides, and amino acids, particularly in the wild-type PAK strain. Moreover, the volcano plots revealed that lipids and the associated metabolites were the most significantly perturbed features with 4 mg/liter polymyxin B at 1 and 4 h in both strains (Fig. 2). It is notable that the intracellular levels of two intermediates responsible for the synthesis of UDP-4-amino-4-deoxy-L-arabinose (UDP-L-Ara4N) were significantly elevated by polymyxin B in strain PAK, but not in strain PAK*pmrB6* (Fig. 2 and Table 1). Moreover, our metabolomics results also showed that 4 mg/liter polymyxin B induced dramatic depletions of nucleotides (27.8%; 10 out of 36 nucleotides) in PAK only at 1 h, but no significant perturbations at 4 h. However, nucleotides were not significantly changed in PAK*pmrB6* due to polymyxin B treatment at 4 mg/liter over 24 h (Fig. 1B and 2 and Table 1).

**TABLE 1:**
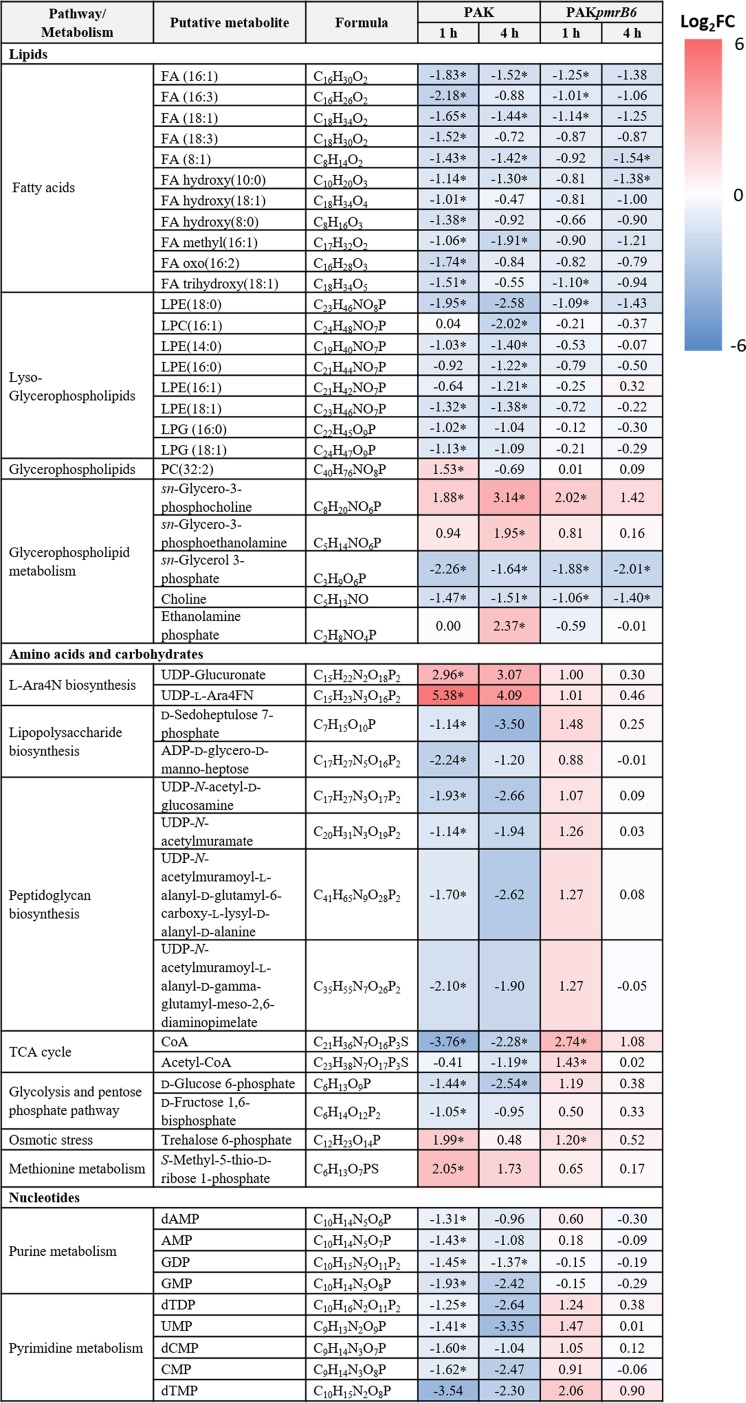
Metabolic perturbations of lipid, amino acid, and nucleotide metabolites in *P. aeruginosa* PAK and PAK*pmrB6* in response to 4 mg/liter polymyxin B at 1 and 4 h^*a*^

a The values for *P*. *aeruginosa* PAK and PAK*pmrB6* strains at 1 and 4 h are shown. The values and whether metabolite level decreased (blue) or increased (red) are shown. A heatmap is shown to the right of the table with the log_2_ fold change (FC) values. Asterisks after values indicate significant change in the abundance of metabolites (>1.0 log_2_ fold change, *P* < 0.05, and FDR < 0.05) (Student’s *t* test).

10.1128/mSystems.00149-18.5 DATA SET S3 (A) Significant metabolites identified in strains PAK and PAK*pmrB6* in response to 4 mg/liter polymyxin B at 1 and 4 h. (B) Significant metabolites identified in strains PAK and PAK*pmrB6* in response to 8× MIC polymyxin B at 1 h. Download Data Set S3, XLSX file, 0.1 MB. Copyright © 2019 Han et al. 2019 Han et al. This content is distributed under the terms of the Creative Commons Attribution 4.0 International license.

### Polymyxin B induced differential transcriptomic changes between polymyxin-susceptible and -resistant P. aeruginosa strains.

**FIG 3:**
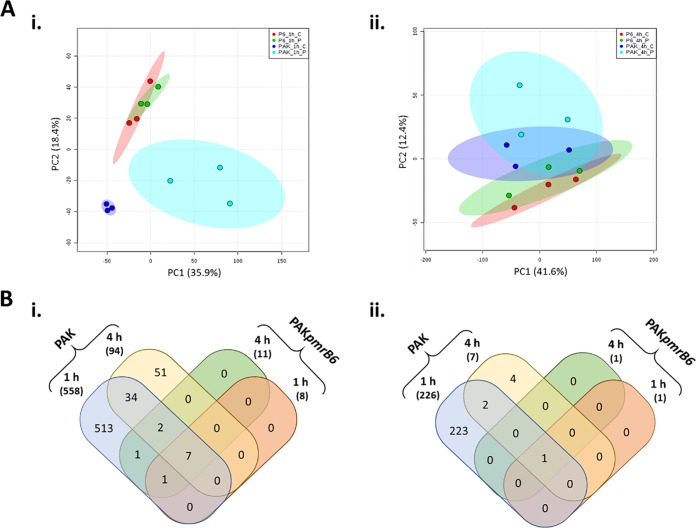
Transcriptomic changes in strains PAK and PAK*pmrB6* in response to polymyxin B (PMB). (A) PCA score plots of the first two components indicate transcriptomic changes in strains PAK and PAK*pmrB6* with the treatment of 4 mg/liter PMB at 1 h (i) and 4 h (ii). Each data set represents three biological replicates of the two strains with and without PMB treatment. The values for untreated PAK (blue), PMB-treated PAK (cyan), untreated PAK*pmrB6* (red), and PMB-treated PAK*pmrB6* (green) are shown. (B) Venn diagrams show the numbers of upregulated genes (i) and downregulated genes (ii) in response to 4 mg/liter PMB in strains PAK and PAK*pmrB6* (fold change > 2, FDR < 0.05).

Polymyxin B at 4 mg/liter also induced markedly different transcriptomic responses between strains PAK and PAK*pmrB6* (Fig. 3). The PCA score plots revealed that 4 mg/liter polymyxin B successfully distinguished the treated samples from those of untreated PAK at 1 h; however, no significant difference was detected at 4 h (Fig. 3A). Notably, polymyxin B treatment led to 558/226 (up/down) differentially expressed genes (DEGs) in strain PAK at 1 h, which sharply decreased to 94/7 at 4 h (Fig. 3B) (FC > 2; false-discovery rate [FDR] < 0.05). Our results demonstrated that bacterial responses to polymyxin B were rapid in polymyxin-susceptible PAK. In the polymyxin-resistant PAK*pmrB6* strain, a minimal transcriptomic response was induced by 4 mg/liter polymyxin B, with only 8/1 (up/down) and 11/1 (up/down) DEGs observed at 1 and 4 h, respectively (Fig. 3). Even in the absence of polymyxin B treatment, a number of different DEGs (40/4) were observed between the wild-type PAK and the *pmrB* mutant PAK*pmrB6* (Table S2). Notably, transcriptional regulators PA4581-4585, PmrAB (PA4776-4777), and the regulated *arnBCADTEF-pmrE* operon (PA3552-3558), spermidine biosynthesis (PA4773-4774), ferrous transfer (PA4357-4359), ABC transporter (PA3396-3399), as well as heme biosynthesis (PA0510-0515) were all significantly upregulated (FC > 2) in polymyxin-resistant PAK*pmrB6* compared to polymyxin-susceptible PAK. Conversely, in PAK*pmrB6*, the arginine biosynthesis gene cluster (PA5171-5173) was downregulated (FC < −2) compared to the wild-type strain. Our transcriptomic data also showed significantly increased expression of *crpA* in PAK*pmrB6* than in PAK, which is known to play a role in high-level polymyxin resistance in P. aeruginosa (27).

10.1128/mSystems.00149-18.7 TABLE S2 Transcriptomic changes in strain PAK*pmrB6* compared to strain PAK in the absence of polymyxin B. NA indicates that gene names are not available in the *Pseudomonas* Genome Database. Download Table S2, DOCX file, 0.2 MB. Copyright © 2019 Han et al. 2019 Han et al. This content is distributed under the terms of the Creative Commons Attribution 4.0 International license.

### Polymyxin B induced alterations in lipid profiles and the related metabolites.

**FIG 4:**
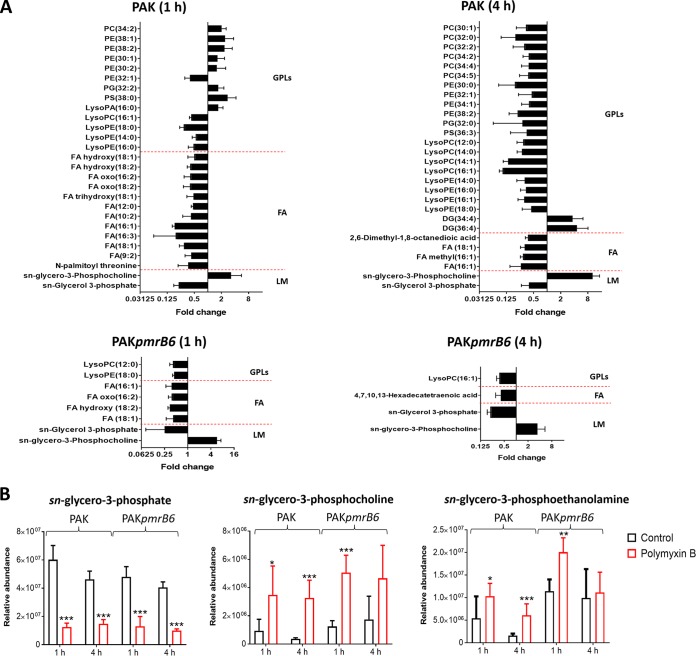
Perturbations of lipids and the related metabolites in strains PAK and PAK*pmrB6* in response to 4 mg/liter polymyxin B at 1 and 4 h. (A) Significantly perturbed lipids of major classes in strains PAK and PAK*pmrB6* following treatment with polymyxin B (4 mg/liter) at 1 and 4 h. Fold change > 2, *P* < 0.05, FDR < 0.05 (Student’s *t* test). Lipids were analyzed using a C8 RPLC method. Lipid names were putatively assigned based on accurate mass. GPLs, glycerophospholipids; FA, fatty acids; LM, lipid metabolism. (B) Depletion of *sn*-glycero-3-phosphate and elevations of *sn*-glycero-3-phosphocholine and *sn*-glycero-3-phosphoethanolamine in strains PAK and PAK*pmrB6* due to 4 mg/liter polymyxin B treatment at 1 and 4 h. Statistical significance (Student's *t* test) is indicated by asterisks as follows: ∗, *P* < 0.05; ∗∗, *P* < 0.01; ∗∗∗, *P* < 0.001.

Polymyxin B treatment at 4 mg/liter significantly perturbed the relative abundance of lipids and the related metabolites in both PAK and PAK*pmrB6* strains mainly at 1 and 4 h (Data Set S3A). Specifically, a number of fatty acids containing 8 to 18 carbons were significantly depleted by polymyxin B treatment in both strains at 1 and 4 h (Table 1), whereas the dramatic depletion of lysophospholipids (phospholipids that have lost one fatty acyl chain from di-acyl phospholipids), including lysophosphatidylethanolamine (LPE), lysophosphatidylcholine (LPC), and lysophophatidylglycerol (LPG), was mainly observed in strain PAK after polymyxin B treatment (4 mg/liter) at both 1 and 4 h (Table 1). Intriguingly, further analysis through RPLC revealed that in PAK after 4 mg/liter polymyxin B treatment, the levels of di-acyl phospholipids, in particular PC, PE, PG. and phosphatidylserines (PS) were dramatically increased at 1 h but decreased at 4 h (Fig. 4A). However, 4 mg/liter polymyxin B did not induce significant changes in the levels of phospholipids and lyso-phospholipids in PAK*pmrB6* (Fig. 4A). Moreover, it is notable that polymyxin B at 4 mg/liter significantly altered the levels of specific metabolites associated with phospholipid metabolism in both strains. Specifically, *sn*-glycerol-3-phosphate (an important precursor in phospholipid synthesis) was depleted (FC = −3.1 to −4.8) in both PAK and PAK*pmrB6* strains with polymyxin B treatment (4 mg/liter) at 1 and 4 h (Table 1 and Fig. 4B) (28). Consistently, its upstream substrates, *sn*-glycerol-3-phosphocholine (FC = 3.7 to 8.8) and *sn*-glycerol-3-phosphoethanolamine (FC = 1.9 to 3.9), accumulated in both strains in response to 4 mg/liter polymyxin B (Fig. 4B). Moreover, the decreased levels of choline (FC = −2.1 to −2.8) which is a by-product of *sn*-glycerol-3-phosphocholine degradation were observed in both strains after 4 mg/liter polymyxin B treatment for 1 and 4 h (Table 1) (28, 29).

**FIG 5:**
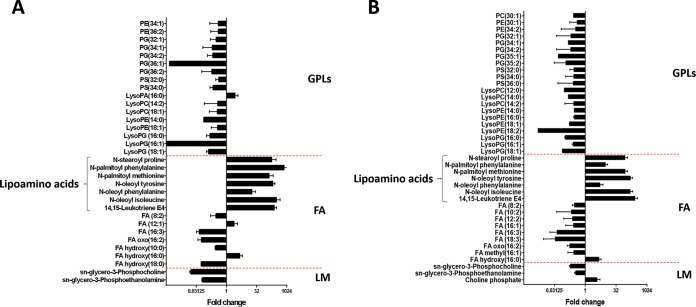
Significant lipid perturbations in response to 8× MIC polymyxin B at 1 h in strain PAK (8 mg/liter) (A) and strain PAK*pmrB6* (128 mg/liter) (B). Fold change > 2, *P* < 0.05, FDR < 0.05 (Student’s *t* test). Lipids were detected using an RPLC method and putatively identified based on the accurate mass. GPLs, glycerophospholipids; FA, fatty acids; LM, lipid metabolism.

The effect of 8× MIC polymyxin B on lipid profiles of both PAK (8 mg/liter) and PAK*pmrB6* (128 mg/liter) strains was also investigated (Fig. 5 and Data Set S3B). Notably, in addition to the dramatic decrease in the levels of most phospholipids and fatty acids, a number of features putatively annotated as amino acid-containing fatty acids [e.g., *N*-oleoyl tyrosine/phenylalanine/(iso)leucine, *N*-palmitoyl methionine/phenylalanine, and *N*-stearoyl proline] were significantly enriched (FC = 5.4 to 802.8) by polymyxin B at 8× MIC in both strains at 1 h (Fig. 5). It is interesting that polymyxin B at 4 mg/liter elevated the levels of phospholipids and the associated precursors (*sn*-glycerol-3-phosphocholine and *sn*-glycerol-3-phosphoethanolamine) in strain PAK at 1 h (Fig. 5); their levels were dramatically decreased by polymyxin B at 8× MIC in both strains at the same time point (Fig. 5).

### Polymyxin B caused metabolic and transcriptomic changes in central carbon metabolism and stress response pathways.

Polymyxin B (4 mg/liter) differentially altered the levels of metabolites related to central carbon metabolism in both PAK and PAK*pmrB6* strains. Specifically, coenzyme A (CoA), which is associated with the tricarboxylic acid (TCA) cycle and plays an important role in the synthesis of fatty acids (30), was significantly decreased in its relative abundance in strain PAK (FC = −13.5 and −4.9, respectively) but increased in strain PAK*pmrB6* (FC = 6.7 and 2.1, respectively) under polymyxin B treatment (4 mg/liter) at 1 and 4 h (Table 1). Notably, a critical metabolite in both pentose phosphate and glycolysis pathways, D-glucose-6-phosphate, was depleted by polymyxin B (4 mg/liter) in PAK at 1 h (FC = −2.7), but it had no dramatic change in PAK*pmrB6*. It is also known that D-glucose-6-phosphate functions as a substrate in the synthesis of trehalose-6-posphate as well as the formation of UDP-glucose (31, 32). In our study, the levels of trehalose-6-phosphate were significantly increased in response to 4 mg/liter polymyxin B in both PAK and PAK*pmrB6* at 1 h (FC = 4.0 and 2.3, respectively) (Fig. 2 and Table 1).

**FIG 6:**
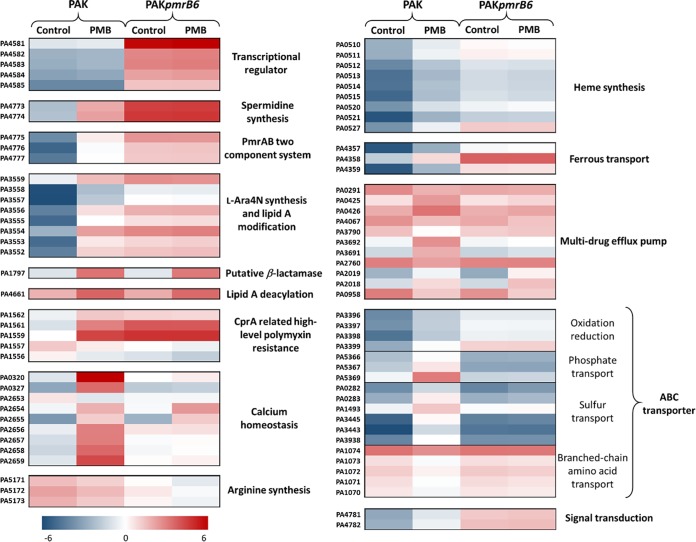
Heatmaps reveal significantly affected pathways by 4 mg/liter polymyxin B (PMB) in strains PAK and PAK*pmrB6* at 1 h using transcriptomics. Gene names are described under P. aeruginosa PAO1 homologues. Transcriptomic expression counts were normalized by sum and transformed to log_2_ scale. Decreased gene expression (blue) and increased gene expression (red) (fold change > 2; FDR < 0.05) are indicated.

The PmrAB-regulated *speDE* (PA4773-4774) operon is responsible for spermidine synthesis, and our transcriptomics data showed its upregulation (FC of 19.4 for *speD* and 22.0 for *speE*) in the PAK strain after 1-h polymyxin B treatment (Fig. 6) (33). Our metabolomic result revealed that a key by-product of spermidine production, *S*-methyl-5-thio-D-ribose-5-phosphate (*S*-MTRP) was dramatically enriched (FC = 4.1) in PAK in response to 4 mg/liter polymyxin B at 1 h (Table 1) (34). In addition to *speDE*, other PmrAB-mediated genes related to stress responses, such as *feoABC* (PA4359 to PA4357) and *ssuAC* (PA3445 and PA3443) (35, 36), were also upregulated (FC = 11.3 to 90.5) in strain PAK after 4 mg/liter polymyxin B treatment for 1 h (Fig. 6). The role of multidrug efflux pumps in polymyxin resistance remains unclear, but it is interesting to note in PAK the significant upregulation of *mexAB* (PA0425-0426) and *mexXY* (PA2019-2018) (FC > 2) and downregulation of outer membrane porins (PA0291, PA0958, PA2760, PA3790, and PA4067) (FC < −2) after 4 mg/liter polymyxin B treatment at 1 h (Fig. 6).

### Metabolic perturbations in lipopolysaccharide and peptidoglycan synthesis.

**FIG 7:**
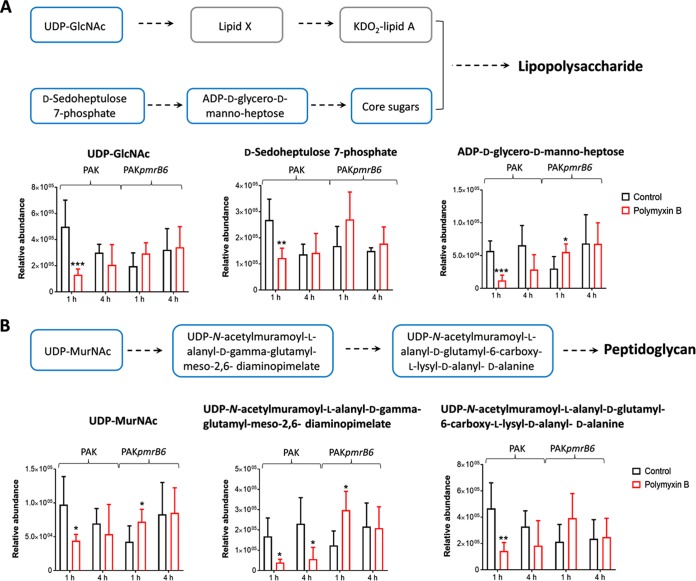
Differential alterations of metabolites associated with synthesis of lipopolysaccharide (A) and peptidoglycan (B) in strains PAK and PAK*pmrB6* in response to polymyxin B treatment (4 mg/liter) at 1 and 4 h. Blue boxes in the flow charts indicate that the metabolites were differentially changed, while gray boxes indicate that the metabolites were not detected by either the HILIC or RPLC method. Statistical significance (Student’s *t* test) is indicated by asterisks as follows: ∗, *P* < 0.05; ∗∗, *P* < 0.01; ∗∗∗, *P* < 0.001.

Polymyxin B at 4 mg/liter also significantly perturbed lipopolysaccharide (LPS) and peptidoglycan syntheses at 1 and 4 h. An important precursor for LPS and cell wall biosynthesis, UDP-*N*-acetyl-D-glucosamine (UDP-GlcNAc) was significantly decreased in strain PAK after polymyxin B treatment (4 mg/liter) at 1 and 4 h (FC = −3.8 and −6.3, respectively) (Fig. 7). Similarly, two intermediates associated with the generation of core sugars of LPS, D-sedoheptulose-7-phosphate (FC = −2.2 and −11.3, respectively) generated from the pentose phosphate pathway (PPP) and ADP-D-glycero-D-manno-heptose (FC = −4.7 and −2.3, respectively) were also significantly depleted by 4 mg/liter polymyxin B at 1 and 4 h (37). In contrast, the relative concentrations of these metabolites were slightly increased (FC = 1.8 to 2.8) in the polymyxin-resistant PAK*pmrB6* strain with polymyxin B treatment at 1 h.

Our results also showed that polymyxin B differentially affected cell wall biosynthesis between the paired polymyxin-susceptible and -resistant strains. In detail, the relative abundance of a major precursor of peptidoglycan synthesis, UDP-*N*-acetylmuramic acid (UDP-MurNAc) was obviously decreased in strain PAK (FC = −3.8 and −6.3, respectively) due to 4 mg/liter polymyxin B treatment at 1 and 4 h but increased in strain PAK*pmrB6* (FC = 2.1) at 1 h. Consistently, the levels of two intermediates in the cell wall synthesis pathway, UDP-*N*-acetylmuramoyl-L-alanyl-D-gamma-glutamyl-*meso*-2,6-diaminopimelate (FC = −4.3 and −3.7, respectively) and UDP-*N*-acetylmuramoyl-L-alanyl-D-glutamyl-6-carboxy-L-lysyl-D-alanyl-D-alanine (FC = −3.2 and −6.1, respectively), were significantly decreased in PAK, while elevated in PAK*pmrB6* (FC = 2.4) with polymyxin B treatment (4 mg/liter) (Fig. 7).

### Metabolic and transcriptomic perturbations in lipid A remodelling.

**FIG 8:**
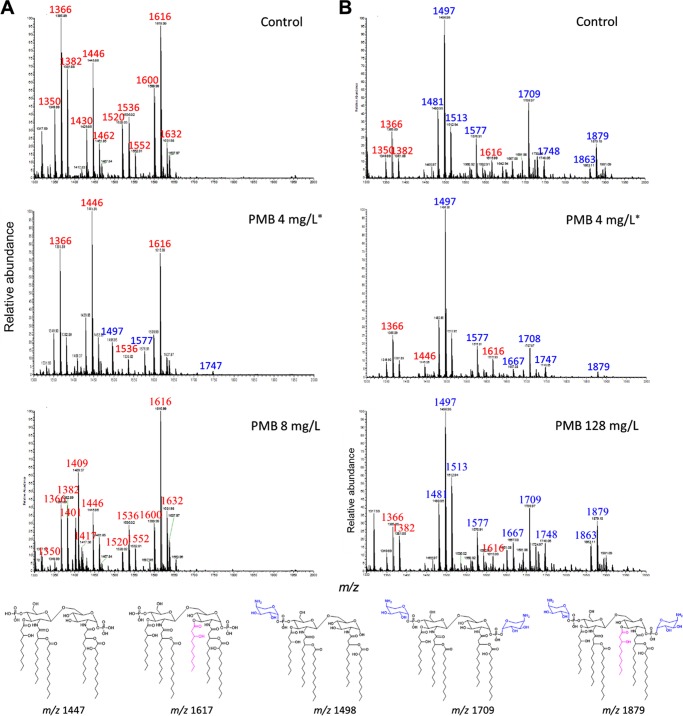
Lipid A analysis of strains PAK (A) and PAK*pmrB6* (B) with and without polymyxin B (PMB) treatment for 1 h. The lipid A samples were analyzed by LC-MS/MS in negative ion mode. The red and blue numbers in the mass spectra indicate wild-type and L-Ara4N-modified lipid A, respectively. In detail, peaks at *m/z* 1350, 1366, 1382, 1430, 1446, and 1462 correspond to penta-acylated lipid A with the loss of one hydrogen ([M − H]^−^), while peaks at *m/z* 1520, 1536, 1552, 1600, 1616, and 1632 represent hexa-acylated lipid A with an *R*-3-hydroxydecanoate at position 3 compared to penta-acylated forms. The major peaks at *m/z* 1481, 1497, and 1513 in the right panel of the mass spectra indicate dephosphorylated penta-acylated lipid A with the addition of an L-Ara4N group. Other peaks at *m/z* 1577, 1667, 1709, 1748, 1863, and 1879 represent lipid A modified with one or two L-Ara4N groups. Three abnormal peaks at *m/z* 1401, 1409, and 1417 in the bottom left mass spectra possibly correspond to doubly charged Kdo2-lipid A. The asterisks in the figure indicate that the lipid A remodelling in response to 4 mg/liter polymyxin B in PAK and PAK*pmrB6* strains was described in reference 39.

The most common mechanism of polymyxin resistance in P. aeruginosa is the modification of lipid A phosphate groups with positively charged L-Ara4N (11). From our untargeted metabolomics study, two key intermediates related to L-Ara4N biosynthesis, UDP-glucuronate (FC = 7.8) and UDP-4-deoxy-4-formamido-L-arabinose (UDP-L-Ara4FN) (FC = 41.6) were significantly enriched in strain PAK after 4 mg/liter polymyxin B treatment even at 1 h (Table 1). Consistently, extensive modifications of lipid A phosphates with an L-Ara4N group (peaks at *m/z* 1497, 1577, and 1747) rapidly occurred even within 1 h in response to polymyxin B (4 mg/liter) (Fig. 8). Transcriptomic analysis of the DEGs from strain PAK treated by 4 mg/liter polymyxin B for 1 h revealed the upregulation of the *arnBCADTEF* (PA3552-3559) operon (FC = 11.8 to 47.8; FDR < 0.05) and the PmrAB two-component regulatory system (TCR) (FC = 19.8 for *pmrA* and 42.2 for *pmrB*) (Fig. 6). This upregulation activated the L-Ara4N biosynthesis pathway which was responsible for the enriched abundance of UDP-glucuronate (FC = 7.8) and UDP-L-Ara4FN (FC = 41.6) (Table 1) as well as the L-Ara4N modifications of lipid A (Fig. 8) (38). In addition, in our recent study, we reported an upregulation of *pagL* (PA4661) (FC > 3) in both PAK and PAK*pmrB6* strains in response to 4 mg/liter polymyxin B over 24 h, which resulted in the deacylation of lipid A in both strains (39). The relationships of lipid A deacylation with polymyxin resistance have been confirmed using neutron reflectometry (39). Moreover, the effect of polymyxin B at a higher concentration (i.e., 8× MIC) on the lipid A modification pathway in both PAK (8 mg/liter) and PAK*pmrB6* (128 mg/liter) was also investigated. Remarkably, in L-Ara4N synthesis, UDP-glucuronate and UDP-L-Ara4FN were not significantly altered by polymyxin B at 8× MIC in either strain PAK or PAK*pmrB6* (Data Set S3B). Accordingly, unlike 4 mg/liter polymyxin B treatment, 8× MIC polymyxin B did not cause dramatic lipid A modifications (Fig. 8).

## DISCUSSION

**FIG 9:**
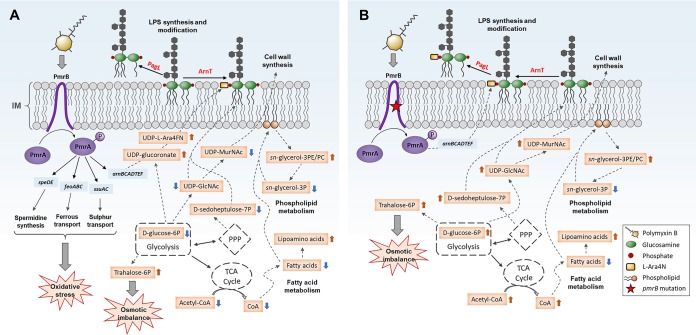
Overview of metabolic and transcriptomic responses of the polymyxin-susceptible PAK strain (A) and polymyxin-resistant PAK*pmrB6* strain (B) to polymyxin B treatment. In strain PAK, polymyxin B (4 mg/liter) significantly upregulated the PmrAB regulatory system and related genes, resulting in the increased L-Ara4N synthesis. Meanwhile, polymyxin B induced severe oxidative stress and osmotic imbalance and significantly decreased metabolite levels related to LPS and peptidoglycan synthesis in the wild-type PAK strain. In contrast, in strain PAK*pmrB6*, 4 mg/liter polymyxin B did not activate the PmrAB system and related genes; therefore, the L-Ara4N synthesis pathway was not affected. Moreover, polymyxin B significantly elevated the metabolites associated with LPS and peptidoglycan synthesis pathways in the polymyxin-resistant PAK*pmrB6* strain. In addition, polymyxin B induced osmotic stress in both PAK and PAK*pmrB6* strains. It is also notable that in both strains, a high concentration of polymyxin B (8× MIC) significantly decreased fatty acid and phospholipid levels but increased lipoamino acid levels. The red and blue arrows indicate that the levels of metabolites were significantly increased and decreased, respectively (fold change > 2, *P* < 0.05, FDR < 0.05, Student’s *t* test).

For the first time, the present study elucidated different metabolic and transcriptomic changes between paired polymyxin-susceptible and -resistant P. aeruginosa strains associated with both polymyxin killing and the development of resistance (Fig. 9). The current literature supports the idea that polymyxins affect the OM physical integrity and lead to phospholipid exchange (4). As predicted, polymyxin B treatment (4 mg/liter) led to profound perturbations of several key lipid metabolites mainly in the wild-type PAK strain (Fig. 4A), possibly due to the membrane-targeted killing by polymyxins (5). Our metabolomics data indicated that, after treatment with a clinically relevant concentration of polymyxin B, the decreased level of lyso-phospholipids in strain PAK was probably due to the suppression of phospholipid degradation, and therefore resulted in an accumulation of phospholipids at 1 h. As the decreased levels of fatty acids and *sn*-glycerol-3-phosphate might contribute to the inhibition of phospholipid synthesis (40), a decreased level of phospholipids observed in PAK at 4 h supports this hypothesis. Our results are consistent with the metabolomic findings for Acinetobacter baumannii that colistin treatment significantly decreased the OM lipid levels and disrupted membrane asymmetry (18, 20, 41). Importantly, this finding supports the notion proposed in our recent study that decreased phospholipid levels observed in the *pmrB* mutants PAK*pmrB6* and PAK*pmrB12* possibly play important roles in polymyxin resistance in P. aeruginosa (42). On the other hand, a high concentration of polymyxin B (8× MIC) substantially disrupted the OM phospholipids and their synthesis in both P. aeruginosa PAK and PAK*pmrB6* (Fig. 5). Therefore, we speculate that the dramatically elevated lipoamino acid levels may contribute to the stabilization of the OM by counteracting the negative charge of LPS (43), thereby diminishing the interaction with positively charged polymyxin molecules. However, the precise biological functions of lipoamino acids in Gram-negative bacteria and the relationship with polymyxin resistance are not clear and are under investigation in our laboratory.

In addition to disorganizing the OM, our results indicated that polymyxin B also interfered with the biosynthesis of both LPS and cell wall in the wild-type PAK strain (Fig. 7); this finding is consistent with our previous study in A. baumannii (18). However, the different metabolic changes in strain PAK*pmrB6* suggested that the polymyxin-resistant strain responded to polymyxin treatment by promoting the biosynthesis of LPS and peptidoglycan, possibly to cope with the cell envelope damages by polymyxins. The central carbon metabolism plays an essential role in generating metabolic precursors in bacteria (30). Our metabolomics data showed different perturbations in the levels of CoA and D-glucose-6-phosphate between the polymyxin-susceptible PAK strain and the polymyxin-resistant PAK*pmrB6* strain (Table 1). Notably, elevated trehalose-6-phosphate levels were evident after 4 mg/liter polymyxin B treatment at 1 h. As the dephosphorylated form trehalose is a well-known osmoprotectant when bacterial cells are under osmotic stress (44), the elevated trehalose-6-phosphate levels suggest that 4 mg/liter polymyxin B induced osmotic imbalance in both polymyxin-susceptible and -resistant strains at 1 h.

Our transcriptomics results indicated that the dramatically increased expression of PmrAB-regulated *speDE*, *feoABC*, and *ssuAC* operons as well as several ABC transporters in strain PAK was related to oxidative stress due to polymyxin B treatment (Fig. 6). Spermidine has been reported to stabilize and protect the bacterial OM against antibiotic and oxidative damage (33). The upregulation of the *speDE* operon in strain PAK*pmrB6* was possibly related to oxidative stress caused by polymyxin B and contributed to polymyxin resistance (33, 45). Consistently, our recent study revealed perturbed spermidine levels and the methionine salvage cycle in the *pmrB* mutants compared to the wild-type PAK strain even without polymyxin treatment (42). The FeoABC transporter is a well-conserved system in the transport of ferrous iron in bacteria (46). It is known that *feoABC* plays an important role in promoting bacterial growth in response to low iron and Mg^2+^ concentrations (35). Therefore, it was very likely that the expression of the *feoABC* operon played a key role in the bacterial survival from polymyxin B treatment. Our results also suggested that polymyxin B treatment potentially led to sulfate starvation, while the upregulation of the *ssu* operon helped to counter the oxidative stress (36). In addition, the significant upregulation of several genes regulated by ParRS (e.g.*, pagL*, PA1797, and *mexXY*) due to polymyxin B treatment in both the wild-type and *pmrB* mutant strains is indicative of the interactions between the two TCRs PmrAB and ParRS (13, 14, 27).

Our correlated metabolomic and transcriptomic data demonstrated, for the first time, that bacterial cells rapidly responded to polymyxins by lipid A modifications, thereby minimizing the interaction and subsequent cellular damage and developing resistance (Fig. 8). This finding is also consistent with the minimal metabolic and transcriptomic perturbations observed in strain PAK*pmrB6* following polymyxin B treatment (4 mg/liter), which was very likely due to the dramatically diminished interaction between polymyxin molecules and L-Ara4N modified lipid A (39). Considering the pharmacokinetics/pharmacodynamics of polymyxins, the 4-mg/liter polymyxin B concentration employed in the present study is higher than the unbound average steady-state concentration (*f*C_ss,avg_) of 1.17 mg/liter (i.e., C_ss,avg_ 2.79 mg/liter × unbound fraction 0.42) in patients with the currently recommended dosage regimens (47); combination therapy should be strongly recommended to minimize any potential emergence of resistance to this important last-line class of antibiotics. Polymyxin B at a super MIC (e.g., 8× MIC) severely damaged bacterial cells and halted the remodelling of their LPS (e.g., adding L-Ara4N to lipid A) (Fig. 8), indicating that both strains were not able to generate polymyxin resistance at super MICs. Unfortunately, for P. aeruginosa isolates with MICs ≥ 0.5 mg/liter, an *f*C_ss,avg_ of 8× MIC is difficult to achieve in patients with the current dosage regimens (47–49).

Collectively, our findings on the complex and dynamic interactions of multiple cellular pathways provide key mechanistic insights into understanding the mechanisms of activity and resistance to polymyxins. This study highlights the urgency of developing rational combination therapy to reduce polymyxin resistance due to rapid lipid A modifications. Our results may also benefit the discovery of much-needed new-generation polymyxins targeting polymyxin-resistant Gram-negative 'superbugs'.

## MATERIALS AND METHODS

### Chemical and reagents.

Polymyxin B was purchased from Sigma-Aldrich (Sydney, New South Wales, Australia). The stock solution of polymyxin B (1 mg/ml) was prepared using Milli-Q water (Millipore Australia, North Ryde, NSW, Australia) and filtered through 0.22-μm syringe filters (Sartorius, Melbourne, VIC, Australia).

### Bacterial strains and culture.

P. aeruginosa PAK and PAK*pmrB6* strains were obtained from the Moskowitz laboratory (Massachusetts General Hospital, MA, USA) (50). The polymyxin B MICs of P. aeruginosa PAK and PAK*pmrB6* strains were determined by broth microdilution (25). Prior to experiments, strain PAK was subcultured on Mueller-Hinton agar plates, while the mutant strain PAK*pmrB6* was subcultured on Mueller-Hinton agar plates containing 4 mg/liter polymyxin B and incubated for 16 to 18 h at 37°C. A single colony was then inoculated into 10 ml of cation-adjusted Mueller-Hinton broth (CaMHB) (Oxoid) and incubated overnight at 37°C with shaking at 150 rpm. The overnight culture was diluted 1:100 into three different reservoirs with 100 ml fresh CaMHB medium and then grown to an optical density at 600 nm (OD_600_) of 0.50 ± 0.02 (∼10^8^ CFU/ml). The bacterial cultures of strains PAK and PAK*pmrB6* were then treated with polymyxin B at 4 mg/liter, 8 mg/liter (PAK), and 128 mg/liter (PAK*pmrB6*) for 1, 4, and 24 h; the untreated bacterial culture served as a control sample.

### Preparation of metabolomics samples.

Cellular metabolites of PAK and PAK*pmrB6* strains were extracted by a previously optimized method with slight modifications (18). Briefly, both treated and untreated samples were collected at 0, 1, 4, and 24 h for metabolite extraction. Bacterial cultures (20 ml) were collected and immediately transferred into 50-ml ice-cold Falcon tubes. Samples were then rapidly quenched in a dry ice/ethanol bath for ∼30 s to stop the metabolic processes and normalized according to OD_600_ at 0.50 ± 0.02 to ensure that the bacterial cell counts were at ∼10^8^ CFU/ml. Cell pellets were then collected from 10 ml normalized culture after centrifugation at 3,220 × *g* at 4 °C for 10 min. After the cell pellets were washed twice with 2 ml ice-cold 0.9% NaCl, they were resuspended in 500 μl chloroform/methanol/water (CMW) (1:3:1 [vol/vol]) containing 1 μM generic internal standards (CHAPS, CAPS, PIPES, and TRIS). A freeze-thaw process was performed three times to lyse the cells and release cellular metabolites. The extracted samples were centrifuged at 3,220 × *g* at 4°C for 10 min, and a 300-μl supernatant was collected, which was followed by a further centrifugation at 14,000 × *g* for 10 min at 4 °C to obtain particle-free supernatants (200 μl) for LC-MS analysis.

### LC-MS analysis of metabolites.

Both hydrophilic interaction liquid chromatography (HILIC) and reversed-phase liquid chromatography (RPLC) coupled to high-resolution mass spectrometry (HRMS) were employed to ensure the detection of both hydrophilic and hydrophobic metabolites. Samples were analyzed on a Dionex U3000 high-performance liquid chromatography system (HPLC) in tandem with a Q-Exactive Orbitrap mass spectrometer (Thermo Fisher) in both positive and negative ion modes with a resolution at 35,000. The HILIC method was described previously in detail (18). Briefly, samples maintained at 4°C were eluted through a ZIC-pHILIC column (5 μm, polymeric, 150 by 4.6 mm; SeQuant, Merck) by mobile phase A (20 mM ammonium carbonate) and mobile phase B (acetonitrile). The gradient started with 80% mobile phase B at a flow rate of 0.3 ml/min and was followed by a linear gradient to 50% mobile phase B over 15 min. The Ascentis Express C8 column (5 cm by 2.1 mm, 2.7 μm) (catalog no. 53831-U; Sigma-Aldrich) was applied in the RPLC method. The samples were controlled at 4°C and eluted by mobile phase A (40% of isopropanol and 60% of Milli-Q water with 8 mM ammonium formate and 2 mM formic acid) and mobile phase B (98% of isopropanol and 2% of Milli-Q water with 8 mM ammonium formate and 2 mM formic acid). The linear gradient started from 100% mobile phase A to a final composition of 35% mobile phase A and 65% mobile phase B over 24 min at 0.2 ml/min. All samples were analyzed within a single LC-MS batch to avoid variations. The pooled quality control samples (QC), internal standards, and total ion chromatograms were assessed to evaluate the chromatographic peaks, signal reproducibility, and stability of analytes. To assist the identification of metabolites, a mixture of ∼600 metabolite standards was analyzed within the same batch.

### Data processing and statistical analyses.

Metabolomics data analyses were performed using mzMatch and IDEOM (http://mzmatch.sourceforge.net/ideom.php) (51). The quantification of each metabolite was based on the chromatogram raw peak height (relative intensity). Univariate and multivariate statistical analyses were conducted using MetaboAnalyst 3.0 (52). Prior to analysis, the data set of relative peak intensity was normalized by the median, log transformed, and auto-scaled. Unsupervised principal-component analysis (PCA) was applied for the analysis of global metabolic profiles, while Student’s *t* test (*P* < 0.05; false-discovery rate [FDR] < 0.05) was used to identify significantly changed metabolites in polymyxin B-treated samples relative to untreated control samples at each time point. Metabolites that showed a fold change of >2 were further analyzed and subjected to metabolic pathway analysis using the KEGG pathway (53), BioCyc (54), and Visualisation and Analysis of Networks containing Experimental Data (VANTED) software (55).

### RNA extraction and analysis of RNA sequencing data.

Polymyxin B-treated and untreated samples (1.5 ml) were collected at 0, 1, 4, and 24 h for RNA extraction. RNAprotect (Qiagen) was used for the sample collection in order to preserve gene expression profiles. RNA was isolated using an RNeasy minikit (Qiagen) in accordance with the manufacturer’s instructions. RNA-Seq was undertaken using Illumina HiSeq (56). RNA-seq data were analyzed according to the methods described previously (39). Briefly, the transcriptome was assembled based on the RNA-Seq data using Trinity RNA-Seq software, and the RNA-Seq reads were aligned according to the genome sequences of P. aeruginosa PAK and PAK*pmrB6* strains using Subread (57). The RNA-Seq data were analyzed using voom and limma linear models through Degust interactive Web-based RNA-Seq visualization software (http://degust.erc.monash.edu) (58).

### Isolation and structural characterization of lipid A.

Lipid A was isolated by mild acid hydrolysis as previously described (59, 60). Briefly, *P. aeruginosa* PAK and PAK*pmrB6* were treated by polymyxin B at 4 mg/liter and 8× MIC for 1 h. The bacterial cell pellets were harvested from 100 ml of normalized culture (OD_600_ = 0.50 ± 0.02) via centrifugation at 3,220 × *g* for 20 min and washed twice with 5 ml PBS. Cell pellets were then resuspended in 4 ml PBS, which was followed by resuspension in 5 ml chloroform and 10 ml methanol to make a single-phase Bligh-Dyer (chloroform/methanol/water, 1:2:0.8 [vol/vol]). After 15-min centrifugation at 3,220 × *g*, the supernatant was removed, leaving LPS in the pellets. After washing once with 5 ml single-phase Bligh-Dyer solvent, the LPS pellets were resuspended in 10.8 ml of hydrolysis buffer (50 mM sodium acetate [pH 4.5] with 1% sodium dodecyl sulfate [SDS]) and homogenized via sonication with a probe tip sonicator (Misonix, USA) at a constant duty cycle (20 s at 50% output). The samples were then incubated in a boiling water bath for 45 min and allowed to cool to room temperature. To extract lipids after hydrolysis, 12 ml of chloroform and 12 ml of methanol were added to the 10.8-ml hydrolysis solution to make a double-phase Bligh-Dyer (chloroform/methanol/water, 1:1:0.9 [vol/vol]). The lower phase containing lipid A was finally collected and dried under nitrogen gas stream. Structural analysis of lipid A was performed using mass spectrometry in negative ion mode on a Q-Exactive Hybrid Quadrupole-Orbitrap mass spectrometer.

### Data availability.

All transcriptomic raw data were deposited in GenBank under accession numbers SRX4714399 to SRX4714440. The metabolomic data set is publicly available at MetaboLights under the study identifier MTBLS751.
