# Supplementary figures and images for: Comparative Metabolomics and Transcriptomics Reveal Multiple Pathways Associated with Polymyxin Killing in Pseudomonas aeruginosa
Source: mSystems. 2019 Jan 8;4(1):e00149-18. doi: 10.1128/mSystems.00149-18 (PMC6325167; doi:10.1128/mSystems.00149-18)

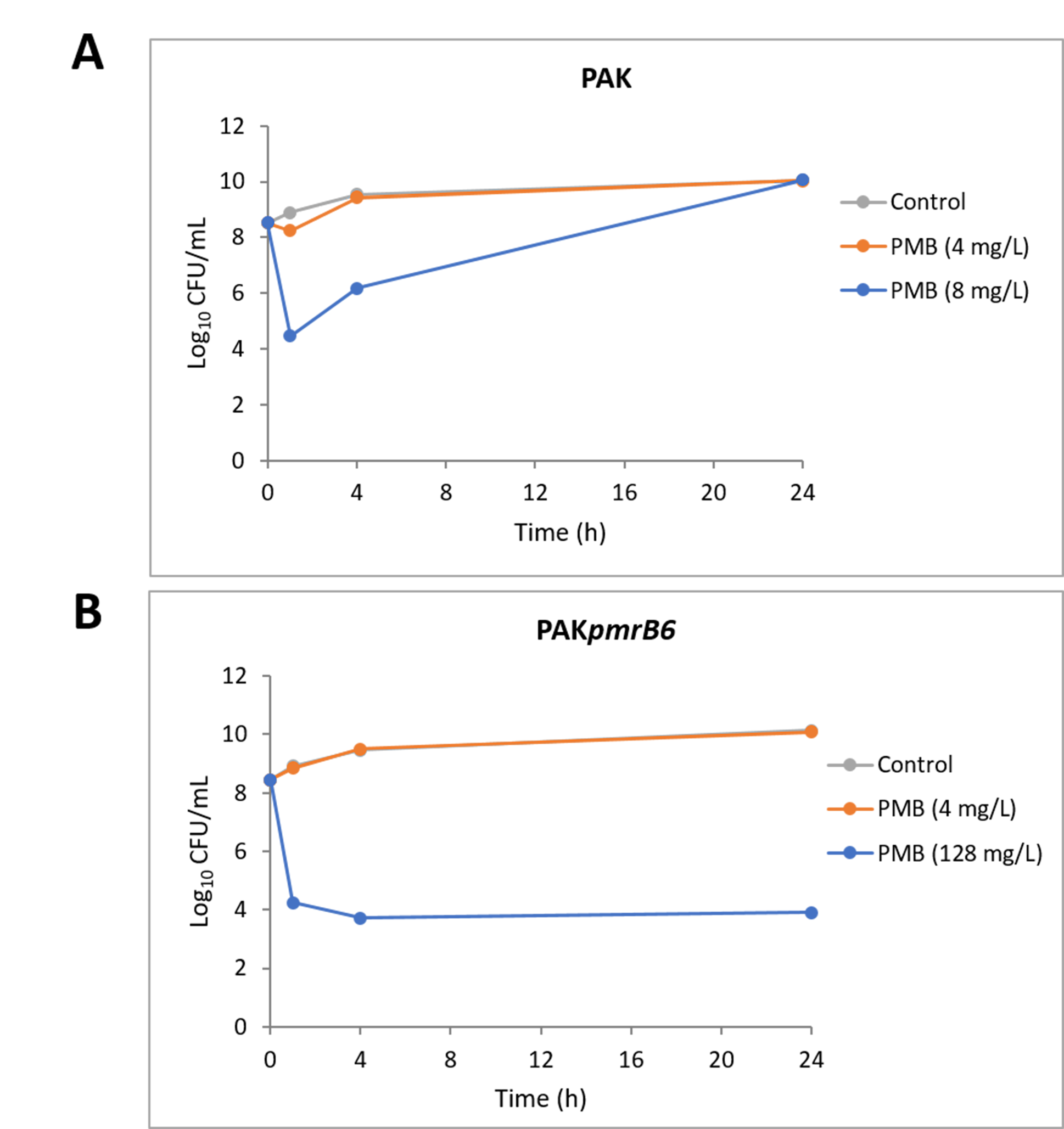

Supplement: FIG S1 [file sys001192312sf7.tif]

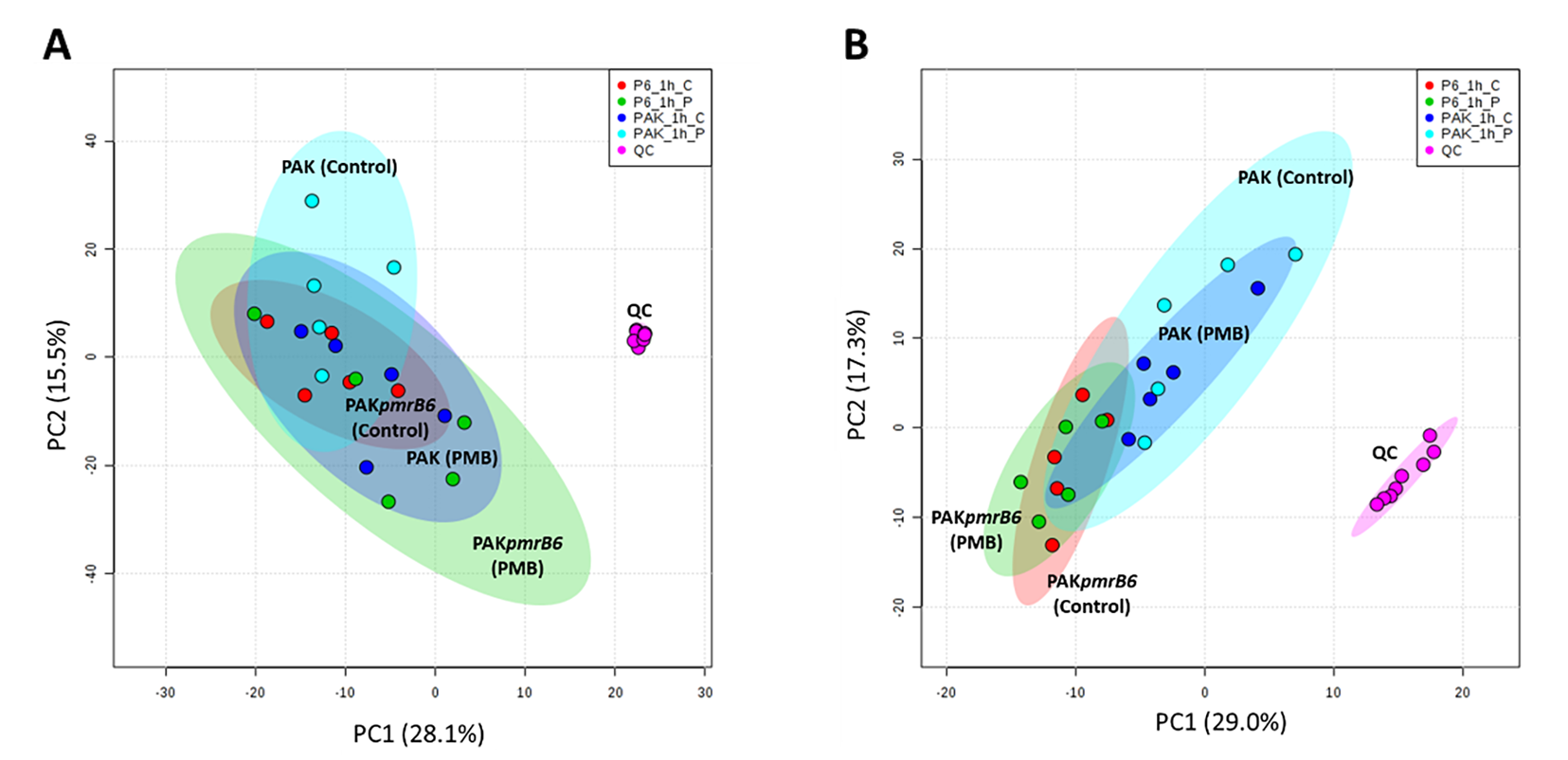

Supplement: FIG S2 [file sys001192312sf6.tif]
