# Supplementary material for: Comparative Metabolomics and Transcriptomics Reveal Multiple Pathways Associated with Polymyxin Killing in Pseudomonas aeruginosa
Source: mSystems. 2019 Jan 8;4(1):e00149-18. doi: 10.1128/mSystems.00149-18 (PMC6325167; doi:10.1128/mSystems.00149-18)
Supplement: TABLE S1 [file sys001192312st5.docx]

**Table S1.** **The median relative standard deviation (RSD) values for all metabolites.** Each dataset was based on five biological replicates measured by HILIC or RPLC methods.

| **HILIC** | **Median RSD (%)** | **RPLC** | **Median RSD (%)** |
| --- | --- | --- | --- |
| PAK_0h | 27.1 | PAK_0h | 30.7 |
| PAK_1h_control | 25.0 | PAK_1h_control | 25.9 |
| PAK_1h_PMB_4mg | 27.5 | PAK_1h_PMB_4mg | 35.0 |
| PAK_4h_control | 30.6 | PAK_4h_control | 30.0 |
| PAK_4h_PMB_4mg | 32.2 | PAK_4h_PMB_4mg | 39.6 |
| PAK_24h_control | 38.3 | PAK_24h_control | 39.5 |
| PAK_24h_PMB_4mg | 33.8 | PAK_24h_PMB_4mg | 35.2 |
| PAK*pmrB6*_0h | 27.0 | PAK*pmrB6*_0h | 27.2 |
| PAK*pmrB6*_1h_control | 24.8 | PAK*pmrB6*_1h_control | 28.8 |
| PAK*pmrB6*_1h_PMB_4mg | 30.5 | PAK*pmrB6*_1h_PMB_4mg | 31.7 |
| PAK*pmrB6*_4h_control | 36.8 | PAK*pmrB6*_4h_control | 31.5 |
| PAK*pmrB6*_4h_PMB_4mg | 27.9 | PAK*pmrB6*_4h_PMB_4mg | 32.6 |
| PAK*pmrB6*_24h_control | 36.8 | PAK*pmrB6*_24h_control | 37.2 |
| PAK*pmrB6*_24h_PMB_4mg | 40.2 | PAK*pmrB6*_24h_PMB_4mg | 36.2 |
| QC_4mg | 10.5 | QC_4mg | 12.9 |
| PAK_1h_control_8MIC | 30.4 |  |  |
| PAK_1h_PMB_8MIC | 36.2 |  |  |
| PAK*pmrB6*_1h_control_8MIC | 25.7 |  |  |
| PAK*pmrB6*_1h_PMB_8MIC | 39.5 |  |  |
| QC_8MIC | 27.0 |  |  |
