# Supplementary material for: Comparative Metabolomics and Transcriptomics Reveal Multiple Pathways Associated with Polymyxin Killing in Pseudomonas aeruginosa
Source: mSystems. 2019 Jan 8;4(1):e00149-18. doi: 10.1128/mSystems.00149-18 (PMC6325167; doi:10.1128/mSystems.00149-18)
Supplement: TABLE S2 [file sys001192312st4.docx]

**Table S2. Transcriptomic changes in PAK*pmrB6* compared to PAK in the absence of polymyxin treatment.** NA, gene names are not available in the *Pseudomonas* Genome Database.

| **Function** | **Locus tag in PRK** | **Locus tag in PAK** | **Locus tag in PAO1** | **Gene name** | **Product (subject)** | **log_2_FC** | **FDR** |
| --- | --- | --- | --- | --- | --- | --- | --- |
| Transcriptional regulator | PRK_00107 | PAK_05048 | PA4581.1 | NA | tRNA-Arg | 7.37 | 3.41E-18 |
|  | PRK_00108 | PAK_05049 | PA4582 | NA | conserved hypothetical protein | 5.79 | 2.81E-20 |
|  | PRK_00109 | PAK_05050 | PA4583 | *rtcB* | conserved hypothetical protein | 6.08 | 2.81E-20 |
|  | PRK_00110 | PAK_05051 | PA4584 | NA | conserved hypothetical protein | 5.68 | 1.66E-17 |
|  | PRK_00111 | PAK_05052 | PA4585 | *rtcA* | RNA 3'-terminal phosphate cyclase | 5.63 | 2.11E-16 |
| Polymyxin resistance | PRK_00326 | PAK_05272 | PA4775 | NA | hypothetical protein | 6.78 | 3.55E-12 |
|  | PRK_00327 | PAK_05273 | PA4776 | *pmrA* | PmrA: two-component regulator system response regulator PmrA | 7.21 | 6.64E-10 |
|  | PRK_00328 | PAK_05274 | PA4777 | *pmrB* | PmrB: two-component regulator system signal sensor kinase PmrB | 5.98 | 3.08E-11 |
|  | PRK_02559 | PAK_01629 | PA3559 | *ywqF* | Probable nucleotide sugar dehydrogenase | 3.37 | 5.01E-09 |
|  | PRK_02560 | PAK_01630 | PA3558 | *arnF* | putative 4-amino-4-deoxy-L-arabinose-phosphoundecaprenol flippase subunit F | 5.48 | 6.01E-08 |
|  | PRK_02561 | PAK_01631 | PA3557 | *arnE* | 4-amino-4-deoxy-L-arabinose-phospho-UDP flippase subunit E | 6.05 | 1.05E-09 |
|  | PRK_02562 | PAK_01632 | PA3556 | *arnT* | undecaprenyl phosphate-alpha-4-amino-4-deoxy-L-arabinose arabinosyl transferase | 6.44 | 7.25E-13 |
|  | PRK_02563 | PAK_01633 | PA3555 | *arnD* | putative 4-deoxy-4-formamido-L-arabinose-phosphoundecaprenol deformylase | 5.91 | 2.11E-10 |
|  | PRK_02564 | PAK_01634 | PA3554 | *arnA* | bifunctional UDP-glucuronic acid decarboxylase/UDP-4-amino-4-deoxy-L-arabinose formyltransferase | 5.8 | 2.91E-12 |
|  | PRK_02565 | PAK_01635 | PA3553 | *arnC* | UDP phosphate 4-deoxy-4-formamido-L-arabinose transferase | 6.12 | 3.08E-11 |
|  | PRK_02566 | PAK_01636 | PA3552 | *arnB* | UDP-4-amino-4-deoxy-L-arabinose-oxoglutarate aminotransferase | 5.99 | 1.47E-13 |
| High-level polymyxin resistance | PRK_04713 | PAK_03790 | PA1562 | *acnA* | aconitate hydratase 1 | 1.49 | 5.72E-03 |
|  | PRK_04714 | PAK_03791 | PA1561 | *aer* | aerotaxis receptor Aer | 5.5 | 7.19E-12 |
|  | PRK_04715 | PAK_03792 | PA1559 | *cprA* | hypothetical protein | 5.47 | 7.25E-13 |
|  | PRK_04717 | PAK_03794 | PA1557 | *ccoN2* | Cytochrome c oxidase, cbb3-type, CcoN subunit | -1.85 | 8.95E-03 |
|  | PRK_04718 | PAK_03795 | PA1556 | *ccoO2* | Cytochrome c oxidase, cbb3-type, CcoO subunit, peptidase S41 | -1.82 | 0.02 |
| Spermidine synthesis | PRK_00324 | PAK_05270 | PA4773 | *speD* | *S*-adenosylmethionine decarboxylase | 7.19 | 2.12E-17 |
|  | PRK_00325 | PAK_05271 | PA4774 | *speE* | spermidine synthase | 7.31 | 1.43E-14 |
| Arginine synthesis | PRK_00736 | PAK_05681 | PA5171 | *arcA* | arginine deiminase | -1.97 | 5.19E-03 |
|  | PRK_00737 | PAK_05682 | PA5172 | *arcB* | ornithine carbamoyltransferase, catabolic | -2.11 | 1.87E-03 |
|  | PRK_00738 | PAK_05683 | PA5173 | *arcC* | carbamate kinase | -2.04 | 3.84E-03 |
| Ferrous transport | PRK_05732 | PAK_04815 | PA4357 | *feoC* | hypothetical protein, Ferrous iron transport protein C | 5.42 | 1.02E-08 |
|  | PRK_05733 | PAK_04816 | PA4358 | *feoB* | probable ferrous iron transport protein B | 5.92 | 1.27E-12 |
|  | PRK_05734 | PAK_04817 | PA4359 | *feoA* | probable ferrous iron transport protein A | 6.5 | 2.43E-09 |
| Signal Transduction | PRK_00333 | PAK_05279 | PA4781 | NA | cyclic di-GMP phosphodiesterase | 4.4 | 8.26E-14 |
|  | PRK_00334 | PAK_05280 | PA4782 | NA | hypothetical protein | 4.36 | 2.35E-15 |
| Heme synthesis | PRK_01660 | PAK_00725 | PA0510 | *nirE* | NirE | 2.84 | 0.02 |
|  | PRK_01661 | PAK_00726 | PA0511 | *nirJ* | heme d1 biosynthesis protein NirJ | 3.14 | 0.03 |
|  | PRK_01662 | PAK_00727 | PA0512 | *nirH* | protein NirH | 3.05 | 0.02 |
|  | PRK_01663 | PAK_00728 | PA0513 | *nirG* | protein NirG | 3.51 | 0.04 |
|  | PRK_01664 | PAK_00729 | PA0514 | *nirL* | heme d1 biosynthesis protein NirL | 3.48 | 0.03 |
|  | PRK_01665 | PAK_00730 | PA0515 | NA | probable transcriptional regulator | 4.02 | 5.72E-03 |
|  | PRK_01670 | PAK_00735 | PA0520 | *nirQ* | regulatory protein NirQ | 3.17 | 0.02 |
|  | PRK_01671 | PAK_00736 | PA0521 | *nirO* | probable cytochrome c oxidase subunit | 4.02 | 0.03 |
|  | PRK_01677 | PAK_00742 | PA0527 | *dnr* | transcriptional regulator Dnr | 5.07 | 7.36E-03 |
| ABC transporter | PRK_02721 | PAK_01791 | PA3396 | *nosL* | NosL protein | 3.2 | 0.02 |
|  | PRK_02722 | PAK_01792 | PA3397 | *frpA* | NosY protein, ABC transporter permease | 3.3 | 0.03 |
|  | PRK_02723 | PAK_01793 | PA3398 | *yxlF* | probable transcriptional regulator | 4.39 | 7.36E-03 |
|  | PRK_02724 | PAK_01794 | PA3399 | NA | hypothetical protein | 4.02 | 0.03 |
